# cDNA and Gene Structure of MytiLec-1, A Bacteriostatic R-Type Lectin from the Mediterranean Mussel (*Mytilus galloprovincialis*)

**DOI:** 10.3390/md14050092

**Published:** 2016-05-11

**Authors:** Imtiaj Hasan, Marco Gerdol, Yuki Fujii, Sultana Rajia, Yasuhiro Koide, Daiki Yamamoto, Sarkar M. A. Kawsar, Yasuhiro Ozeki

**Affiliations:** 1 Department of Life and Environmental System Science, Graduate School of NanoBio Sciences, Yokohama City University, 22-2 Seto, Kanazawa-ku, Yokohama 236-0027, Japan; hasanimtiaj@yahoo.co.uk (I.H.); rajia_bio@yahoo.com (S.R.); yasukoide04@yahoo.co.jp (Y.K.); r-ui@outlook.jp (D.Y.); akawsarabe@yahoo.com (S.M.A.K.); 2 Department of Biochemistry and Molecular Biology, Faculty of Science, University of Rajshahi, Rajshahi 6205, Bangladesh; 3 Department of Life Sciences, University of Trieste, Via Licio Giorgieri 5, Trieste 34127, Italy; mgerdol@units.it; 4 Department of Pharmacy, Faculty of Pharmaceutical Science, Nagasaki International University, 2825-7 Huis Ten Bosch, Sasebo, Nagasaki 859-3298, Japan; yfujii@niu.ac.jp; 5 Department of Natural Science, Varendra University, Rajshahi 6204, Bangladesh; 6 Department of Chemistry, Faculty of Sciences, University of Chittagong, Chittagong 4331, Bangladesh

**Keywords:** bacteriostatic activity, cDNA, gene, innate immunity, mytilectin family, *Mytilus galloprovincialis*, mRNA-sequence, MytiLec-1, R-type lectin

## Abstract

MytiLec is an α-d-galactose-binding lectin with a unique primary structure isolated from the Mediterranean mussel (*Mytilus galloprovincialis*). The lectin adopts a β-trefoil fold that is also found in the B-sub-unit of ricin and other ricin-type (R-type) lectins. We are introducing MytiLec(-1) and its two variants (MytiLec-2 and -3), which both possess an additional pore-forming aerolysin-like domain, as members of a novel multi-genic “mytilectin family” in bivalve mollusks. Based on the full length mRNA sequence (911 bps), it was possible to elucidate the coding sequence of MytiLec-1, which displays an extended open reading frame (ORF) at the 5′ end of the sequence, confirmed both at the mRNA and at the genomic DNA sequence level. While this extension could potentially produce a polypeptide significantly longer than previously reported, this has not been confirmed yet at the protein level. MytiLec-1 was revealed to be encoded by a gene consisting of two exons and a single intron. The first exon comprised the 5′UTR and the initial ATG codon and it was possible to detect a putative promoter region immediately ahead of the transcription start site in the MytiLec-1 genomic locus. The remaining part of the MytiLec-1 coding sequence (including the three sub-domains, the 3′UTR and the poly-A signal) was included in the second exon. The bacteriostatic activity of MytiLec-1 was determined by the agglutination of both Gram-positive and Gram-negative bacteria, which was reversed by the co-presence of α-galactoside. Altogether, these data support the classification of MytiLec-1 as a member of the novel mytilectin family and suggest that this lectin may play an important role as a pattern recognition receptor in the innate immunity of mussels.

## 1. Introduction

Revealing the mechanism of the primordial immunity of marine invertebrates holds a great potential in providing a detailed understanding on their adaptation the marine environment based on evolutionary diversification, about the functioning of the marine ecosystem and can also provide useful information for the improvement of biomonitoring programs. The advent of next generation nucleotide sequencing technologies permitted to study in great detail the transcriptome of non-model marine species, including bivalve mollusks, and also allowed to obtain valuable information about gene-encoded molecules relevant for the functioning of the innate immune system of these organisms [1,2,3,4,5,6]. A recent RNA-sequencing approach applied to the Mediterranean mussel (*Mytilus galloprovincialis*), a worldwide economically-important marine bivalve species, elucidated the presence of several lectins (carbohydrate-binding proteins), that act as pattern recognition receptors (PRRs) [4]. PRRs are evolutionary conserved families of extracellular, membrane-bound, and cytosolic molecules that play significant roles in the primitive part of immune system through the recognition of pathogen-associated molecular patterns (PAMPs) [7], and which cover a particularly important role in invertebrate organisms, which lack an adaptive immune system.

The purification of an α-d-Gal-binding lectin, MytiLec (renamed MytiLec-1 in the present manuscript), from *M. galloprovincialis* has been reported in a previous publication [8]. This 17 kDa polypeptide (with 149 amino acids in its primary structure) contains three tandemly-repeated 50- amino acids sub-domains [9]. The 3-D structure of MytiLec-1 obtained by crystallographic analysis revealed a β-trefoil folding [10], which is also found in the ricin B-sub-unit type (R-type) lectins, a widespread lectin family present in bacteria, plants, and animals. Additionally, the 3-D structure indicated that MytiLec was a non-covalently bound dimer consisting of two polypeptides displaying an α-GalNAc-binding activity in each sub-domain [11], with six α-galactoside-binding sites present in a single molecule. Two d-Gal/GalNAc-binding lectins (named CGL and MTL, respectively) have later been described in two other mussel (suborder *Mytiloida*) species, *Crenomytilus grayanus* and *Mytilus trossulus*, and they were found to possess antimicrobial activities. The deduced amino acids sequence from the cDNA of these lectins indicated that MytiLec-1, CGL, and MTL shared high sequence similarity (with 83%–84% identity) [12,13]. Such a remarkable degree of similarity suggests that the possibility to introduce a novel structural family of lectins which comprises all the MytiLec-like sequences found in bivalves. This viewpoint has been strongly supported by a transcriptome analysis performed in *M. galloprovincialis*, which revealed the presence of two other sequences similar to MytiLec-1, named MytiLec-2 and MytiLec-3 in the same species [4]. Unlike MytiLec-1, CGL, and MTL, MytiLec-2 and -3 contain an additional pore-forming aerolysin [14]-like domain located at the C-terminus of the polypeptide that possibly creates pores into pathogenic organisms, thereby contributing to their killing. This aerolysin-like domain, originally found in bacteria, has been recently shown to display a much broader taxonomic range of distribution, as it was identified in a relatively large number of animal, plant, and fungal proteins, most of them with a cytotoxic function [15].

On the other hand, MytiLec-1 does not have any additional C-terminal domains and only consists of a glycan-binding domain. Nevertheless, the possibility that MytiLec-1 could similarly have a role in the killing and clearance of foreign cells was strongly hinted by the specific induction of apoptosis observed in human Burkitt’s lymphoma cells through multiple pathways. It was demonstrated that these tumor cells express Gb3 glycans that contain α-galactose as a target carbohydrate recognized by this lectin [16]. As a bacteriostatic activity has already been described for CGL and MTL, the evidence of a similar activity in MytiLec-1 could confirm a common physiological role for other members of this novel lectin family.

MytiBase is the result of joint international effort to build a database of transcripts expressed in *M. galloprovincialis* by using expressed sequence tag (EST) libraries, with a particular focus on immune-related sequences. The partial cDNA sequence of MytiLec-1 is present in the database since 2009 (MGC00918, resulting from the assembly of 5 EST sequences) [17]. In the present manuscript we report of the full-length sequence of the cDNA (and deduced polypeptide) of MytiLec-1, providing useful information to gain novel insights into the characteristic immune-related properties of this lectin and of the related sequences MytiLec-2 and 3. The information gathered from the analysis of this sequence can be, to some extent, extended to the other members of the “mytilectin family”, helping to better understand how these molecules function as PRRs and take part in the mussel innate immune response.

To provide highly reliable sequence information, both a cDNA cloning and transcriptome sequencing (RNA-seq) analysis approach were undertaken. Although MytiLec-1, like galectin-1, lacks a signal peptide sequence, we showed that it displays a previously unreported extension of the ORF at the 5′ end of the mRNA sequence. The virtually-translated protein sequence contained a 37 amino acid-long extension at the N-terminus compared to the previously reported sequence of MytiLec-1 [17], possibly corresponding to a non-classical secretion signal. Additionally, we elucidated the structure of the MytiLec-1 gene, which consisted of two exons and one intron, and we preliminarily investigated its expression in mussel tissues, identifying mantle and gills as the preferential sites of production. Furthermore, we observed that this lectin could exert a bacteriostatic activity similar to that of CGL and MTL, further supporting a common function for all the members of the mytilectin family. Altogether, these results support the idea that MytiLec-1 may function as a PRR molecule involved in the innate immunity of mussels.

## 2. Results and Discussion

### 2.1. cDNA Sequence of Mytilec-1 and Virtual Translation of the Polypeptide

**Figure 1:**
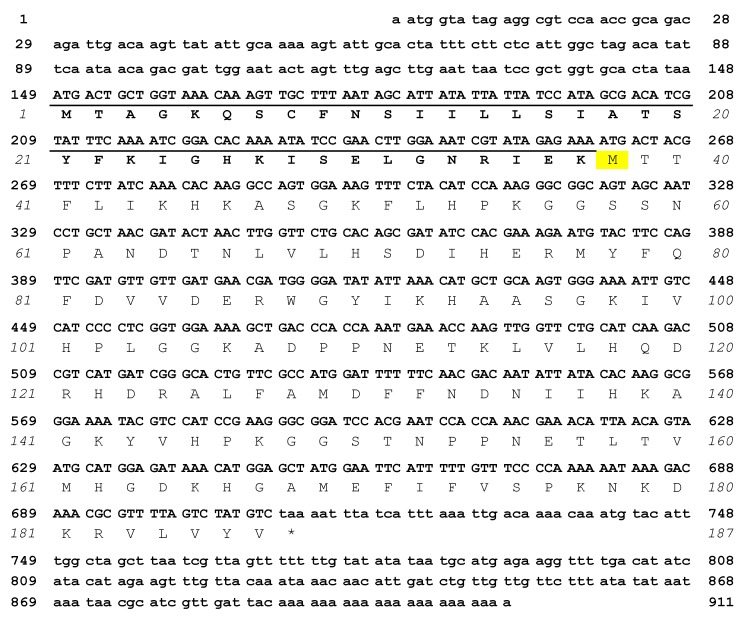
cDNA sequence and deduced amino acid sequence of MytiLec-1. Bold and italic numbers indicate nucleotides and amino acids, respectively. ^38^Met (yellow background) correspond to the first amino acidic residue of the mature MytiLec-1 polypeptide. The alternative N-terminal extension sequence is underlined. The asterisk indicates the stop codon.

The cDNA sequence of MytiLec-1 was identified by Sanger’s dideoxy sequencing method and further confirmed by the analysis of *de novo* assembled RNA-sequencing data (Figure 1). The nucleotide sequence consisted of 911 base pairs (GenBank LC125182.1) [18] and it displayed high homology with the cDNAs encoding lectins from *Mytilus californianus* (GenBank KT695159.1) [19], *M. trossulus* (GenBank KR019779.1) [20], and *Crenomytilus grayanus* (GenBank JQ314213.1) [21]. In particular, the whole nucleotide sequence (450 bps) corresponding to the predicted *M. californianus* polypeptide (149 amino acids) was found to be 100% identical between the two species. This fact was somewhat surprising, as *M. galloprovincialis* and *M. californianus* are two genetically and morphologically distinct species [22]. Such a high degree of similarity at the nucleotide level between these two species could only be explained by introgression, a phenomenon much more spread than originally thought [23], or by species misidentification. On the other hand, the coding nucleotide sequence of MytiLec-1 displayed 88% and 89% identity with MTL and CGL, respectively.

Remarkably, the MytiLec-1 cDNA showed the presence of an additional translatable nucleotide sequence consisting of 111 bps found ahead of the ATG codon corresponding to the amino acidic residue ^1^Met in the other mytilectin sequences deposited in GenBank (Figure 1, underlined). The absence of STOP codons within this region was confirmed by both 5′RACE and by RNA-sequencing. This region possibly encodes a 37 amino acid segment which has no similarity with other sequences deposited in GenBank, as suggested by the absence of significant BLAST hits. The open reading frame is preceded by a 5′UTR region consisting of 148 nucleotides. The 5′UTR of the *M. californianus* lectin mRNA (GenBank KT695159.1) [19] deposited sequence was nearly identical, but much shorter, than that of MytiLec-1, thereby lacking the putative translation start codon observed in MytiLec-1. Furthermore, a recent transcriptome analysis of *M. trossulus* (another species pertaining to the *M. edulis* species complex and closely related to *M. galloprovincialis*) showed that this 5′ extension is also present in the full length mRNA encoding the MTL polypeptide [24].

However, it is noteworthy that the N-terminal sequence of translated MytiLec-1 observed in a previous study was initiated from acetylated-^39^TTFLIK—[8] (Figure 1). Therefore, the alternative N-terminal extension sequence coding ^1^MTAGK—here reported, it does not appear to be part of the mature polypeptide. Different hypotheses could explain this discrepancy: (i) contest-depending leaky scanning [25], which would determine the bypass of the initial ATG codon by the ribosomal 40S subunits in favor of the second ATG codon (encoding ^38^Met); (ii) processing by an unknown extracellular protease, which would cleave off the 37 amino acid-long N-terminal extension, possibly corresponding to a non-canonical secretion signal; (iii) the production of MytiLec-1 isoforms of different length (either starting from ^1^Met or ^38^Met) by the use of different transcription start sites (TSS) under a dispersed promoter model (Section 2.3). The identification of the protein processing system involved in the maturation of MytiLec-1 will also be important to understand the physiological function of this lectin and its orthologous molecules expressed in other species and genera.

### 2.2. Comparison of Deduced Primary Structures among M. galloprovincialis Mytilectins

**Figure 2:**
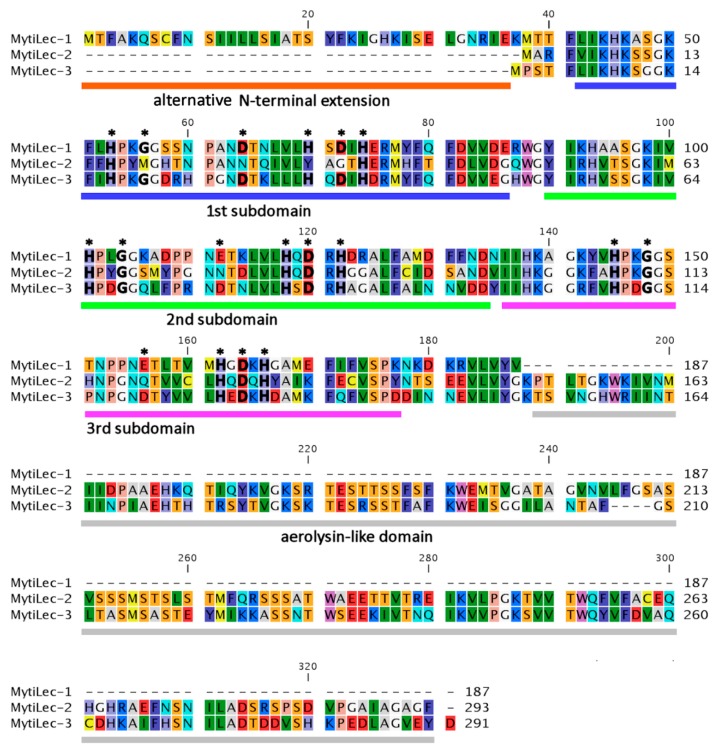
Deduced amino acid sequences of the three members of the mytilectin family in *M. galloprovincialis* (MytiLec-1, -2, and -3). Alternative N-terminal extension (orange), first sub-domain (blue), second sub-domain (green), third sub-domain (pink), and aerolysin-like domain (gray) are shown here. Asterisks denote amino acids essential to ensure the sugar-binding properties [10]. Bold characters within these essential amino acids indicate amino acids that are conserved compared to MytiLec-1.

The primary structure of MytiLec-1 was determined to be 60% and 63% identical with corresponding deduced amino acid sequences of the glycan-binding domain of MytiLec-2 (GenBank KP125931.1) [26] and MytiLec-3 (GenBank KP125932.1) [27], respectively (Figure 2). This result can provide quite a valuable evidence for what concerns the innate immunity-related function of these lectins in mussels. The presence of different types of mytilectins (MytiLec-1, -2, and -3) with different conformations (MytiLec-1 is the prototype having only a glycan-binding domain, whereas MytiLec-2 and -3 combine a lectin and a pore-forming aerolysin-like domain) confirms the presence of a multi-genic “mytilectin family” in this genus of marine bivalve mollusks. Additionally, most of the six essential amino acids (Figure 2, asterisks) that are involved in the recognition and binding of α-*N*-acetyl d-galactosamine [10] in MytiLec-1 were also conserved in MytiLec-2 and -3, suggesting that these variants might possess a glycan-binding activity as well.

Furthermore, the alternative *N*-terminal extension sequence observed in MytiLec-1 is not present in MytiLec-2 nor in MytiLec-3 (dashed lines of MytiLec-2 and -3 in Figure 2). The reason behind this difference will be studied by the comparison of gene structures among prototype (MytiLec-1) and chimera-type (MytiLec-2 and -3) members of the mytilectin family. However, as previously noted, the longer N-terminal region of MytiLec-1 might comprise a non-classical secretion signal (cleaved off upon the release of the peptide in the extracellular space) [4] or may not be translated at all due to context-depending leaky scanning. Different roles of each family member and their regulation in response to external stimuli (e.g., bacterial challenges) will be investigated in the near future to understand the specific function of these lectins as PRR molecules.

### 2.3. The MytiLec-1 Gene Comprises Two Exons

**Figure 3:**
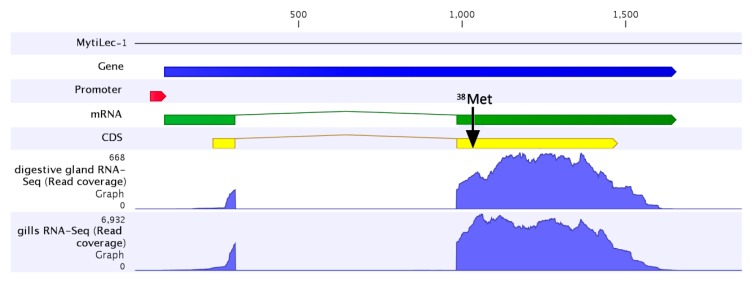
Gene structure of MytiLec-1. The gene and its promoter are indicated as blue and red arrows, respectively. Two exons of the lectin are indicated as a broken green arrow. The coding sequence, including the N-terminal alternative extension, is indicated by a yellow arrow, and the position ^38^Met is indicated by a black arrow. The RNA-seq mapping graphs obtained from digestive gland and gills clearly mark the location of exon/intron junctions.

We investigated the gene structure of MytiLec-1 using the partial genome data available for *M. galloprovincialis*. The gene consisted of two exons and one intron, with a total length of about 1.5 Kb (Figure 3). The first exon comprised the 5′ untranslated region, the alternative initial ATG codon (encoding ^1^Met) and covered the first 23 amino acids of the N-terminal extension of the virtually-translated polypeptide. The remaining part of the MytiLec-1 coding sequence was included in the second exon, together with the 3′UTR and the poly-adenylation site (Figure 3). The two exons were separated by a 676 nucleotides long phase 0 intron. The analysis of the RNA-seq coverage towards the 5′ end of the gene revealed a remarkable feature, as it appears that the coverage drop, which is expected from RNA-seq libraries obtained by mRNA fragmentation protocols, was particularly abrupt, and occurred in a region very close to the nucleotide positions encoding ^1^Met. This could indicate the presence of two or more distinct transcription start sites (TSS) which could be alternatively used under a dispersed promoter model. The most frequently used TSS, based on the coverage analysis, would originate a shorter version of MytiLec-1, missing the initial ATG codon and, therefore, devoid of the initial 37 amino acid residues, resulting in a translated polypeptide fully alignable to the *M. californianus* lectin deposited in GenBank.

However, despite the sharp decrease in 5′ sequencing coverage, a number of reads indicate that a second longer version of the MytiLec-1 transcript is produced, actually corresponding to the sequence reported in Figure 1 and consistent with the cloning results of 5′RACE. Although the precise position of the transcription start sites is dubious, we could detect RNA-seq reads mapped up to position −148 (compared to the initial ATG codon), partially overlapping the putative promoter region, predicted to cover the region between −143 and −192 in the MytiLec-1 locus.

**Figure 4:**
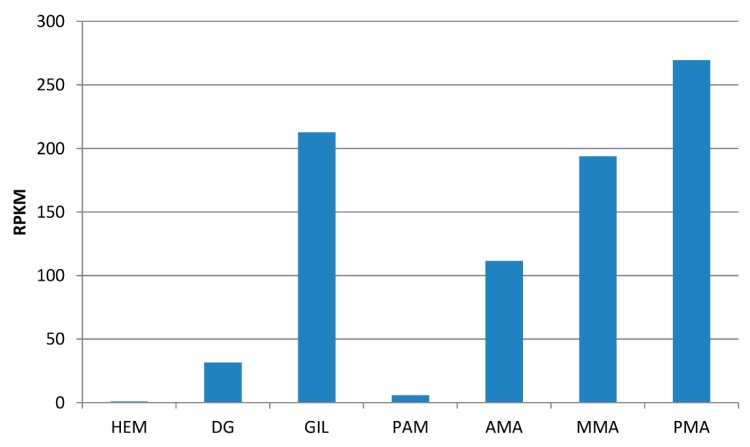
Gene expression of MytiLec-1 in different adult mussel tissues, calculated based on RNA-seq data. Expression levels are given as RPKM (read per kilobase per million mapped reads). HEM: hemocytes; DG: digestive gland; GIL: gills; PAM: posterior adductor muscle; AMA: anterior muscle; MMA: mid mantle; and PMA: posterior mantle.

The RNA-seq mapping graphs obtained from different tissues collected from adult unchallenged mussels (Figure 4) indicated that MytiLec-1 was highly expressed in gills in addition to the mantle (the tissue where MytiLec-1 was originally purified [8]). Within the mantle, MytiLec-1 appeared to be expressed in increasing amounts moving from the anterior towards the posterior region. The observed expression levels were comparatively much lower in the digestive gland and in the posterior adductor muscle, whereas the transcript was barely detectable in hemocytes (RPKM < 1). These results point out a remarkably high level of expression of the MytiLec-1 mRNA in specific tissues (gills and mantle) which are constantly exposed to the outer environment, suggesting that this lectin could be involved in the recognition of glycans exposed on the surface of parasitic and symbiotic microorganisms.

### 2.4. Agglutination of Bacteria by MytiLec-1 Signifies Its Bacteriostatic Activity

**Figure 5:**
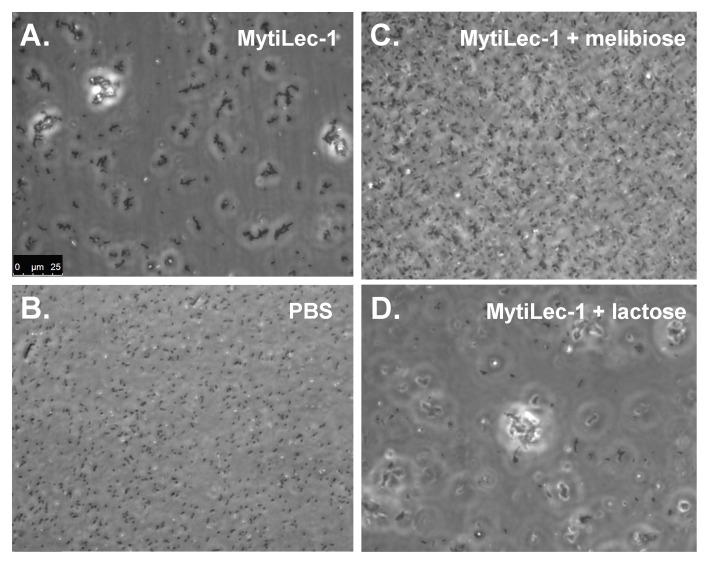
Agglutination of *E. coli* by MytiLec-1. MytiLec-1 (20 μg/mL in (**A**), (**C**), and (**D**)) was applied to the bacteria with 50 mM of melibiose (Galα1-6Glc) and lactose (Galβ1-4Glc), shown in (**C**) and (**D**), respectively. (**B**) (PBS) is the negative control without lectin. The black bar indicates a scale of 25 μm.

MytiLec-1 triggered a strong agglutination of bacteria depending on the strain tested (Figure 4) and suppressed their growth significantly (Table 1). Figure 5 depicts a representative view of the agglutination of *Escherichia coli* by the lectin. The agglutination was specifically inhibited by the co-presence of α-galactoside (Figure 5C) but not by β-galactoside (Figure 5D). Similar results were also observed in other different bacteria (data not shown). In Gram-negative and Gram-positive bacteria, the main target glycoconjugates for several lectins are lipopolysaccharide and the lipoteichoic acid, respectively. These molecules can be considered as PAMPs [28], as they are among the most common targets of PRRs. Both lipopolysaccharide and lipoteichoic acid were reported to contain α-galactose or its derivatives [29,30]. Though the mechanism of bacterial agglutination by MytiLec-1 is still unclear, it is likely that this phenomenon is mediated by the recognition of the aforementioned PAMPs by members of the mytilectin family acting as PRRs.

The bacteriostatic activity of MytiLec-1 is summarized in Table 1. MytiLec-1 (20 μg/mL) significantly reduced the growth of a number of bacteria, similarly to what has been previously described for CGL [12]. Based on the data collected so far, it appears that mytilectins can function either directly as bacterial growth inhibitors or, after agglutination, as modulators of the immune response, by triggering the action of other immune cells and molecules in mussels.

Although the mytilectin family displays unique structural features, as the primary structure of each lectin domain is quite unique, their 3-D structure converges to that of a plant ricin toxin B-subunit (R-type) lectin obtained from castor bean [31]. Further comparative bioinformatics studies of transcriptome and genome data will clarify whether the mytilectin gene family is also found in other mollusks and related phyla or it is taxonomically-restricted to mussels. Among invertebrates, pierisin in the cabbage butterfly (*Pieris rapae*) [32] and CEL-III in sea cucumber (*Cucumaria echinata*) [33] are well known cases of peptides bearing a structural organization similar to that of mytilectins. These two proteins have additional ADP-ribosyltransferase and pore-forming domains connected with an R-type lectin domain and they are able to kill HeLa cells and to trigger the hemolysis of erythrocytes mediated by Gal/GalNAc saccharides. This may suggest that R-type lectins with a PRR function are relatively widespread in invertebrates.

It has been largely documented that the injection of pathogenic microorganisms in *Mytilus* spp. determines the up-regulation of several transcripts encoding lectins, providing an indication that these molecules can act as PRRs in sessile marine bivalves [1]. In previous studies, a survey of lectin and lectin-like transcripts in *M. galloprovincialis* revealed that R-type lectins are also part of this lectin arsenal, in addition to C-type, Con A-type (including galectin), F-type, I-type, SUEL/RBL-type, P-type, and L-type lectins [3]. Many studies support the idea that invertebrate lectins can function as extracellular PRRs, mirroring the function of membrane-bound PRRs, such as Toll-like receptors [6]. The carbohydrate-binding profile of MytiLec-1 suggested that this molecule can recognize α-galactose/galactoside, including N-acetyl α-d-galactosamine, like previously reported for CGL [12,34]. The α-galactoside-binding specificity of this lectin makes it a peculiar mussel PRR, as this property has not been described in the other lectins studied so far in this organism.

The bacteriostatic properties of MytiLec-1, which have been previously reported for other mytilectins (CGL [12] and MTL [13]) were confirmed in this study. Therefore, it seems likely that MytiLec-1 acts as a PRR molecule in a similar fashion to other members of the mytilectin family [4]. The specificity of action and functional overlap between MytiLec-1, -2, and -3 will be investigated in the near future through dedicated studies. One possibility would be that MytiLec-1 has a primary role in recognition and opsonization, whereas MytiLec-2 and -3 are devoted to directly kill bacteria with their aerolysin-like domain. It will be possible to link both the direct (as antibacterial initiator) and indirect (opsonizing activity) effects of MytiLec-1 to its bacteriostatic activity only once the network of molecules interacting with this lectin will be identified in mussels.

The activation of mitogen-activated protein kinases (MAPKs) molecules and the expression of tumor necrotic factor (TNF)-α and NF-κB have already been reported in mussels in response to pathogen challenges [3,35]. Although the specific mechanism of action of MytiLec-1 as a PRR is not yet known, there is evidence that this lectin can activate signal transduction molecules, such as classical MAPKs (MAPK/extracellular signal-regulated kinase (MEK) and extracellular signal-regulated kinase (ERK)), stress-activated MAPK (p38 kinase and c-*Jun* N-terminal kinase (JNK)) and caspase-3/9 with the expression of p21 and tumor necrotic factor (TNF)-α in human Burkitt’s lymphoma cells by the interaction with the target α-galactoside glycan Gb3 [16]. This suggests that MytiLec-1 could potentially activate these intracellular signal transduction pathways in mussels in response to other target glycans. JNK and p38, which were activated by the administration of MytiLec-1 in lymphoma cells, are major players also in the innate immune response of mussel, as they are activated in the hemocytes of *M. galloprovincialis* upon infection with pathogenic microorganisms, including *Vibrio* bacteria, and by high-temperature exposure [36,37]. Galactose residues could serve as target glycans, as they are present in the lipopolysaccharide of various Gram-negative bacteria such as *Vibrio* spp. and *Escherichia coli* [38,39]. Additionally, during the purification process of MytiLec-1, mussel tissues were homogenized with a buffer containing the haptenic sugar d-galactose. The addition of this sugar effectively increased the yield of the lectin compared to a purification performed with a sugar-free homogenizing buffer, indicating that a fraction of the MytiLec-1 peptide might have been bound to the tissue expressing target glycans. Overall, the integration of the genetic and physiological information, together with these functional evidences, suggests that the mytilectin family may play a crucial role in the immune system of bivalves.

## 3. Experimental Design

### 3.1. cDNA Cloning and Sequencing of MytiLec-1

Total RNA was isolated from the mantle and gill of *M. galloprovincialis* (collected in the Tokyo bay, Yokohama, Japan) using TRI reagent^®^ (Molecular Research Center, Cincinnati, OH, USA) and the Direct-zol RNA Mini Prep Kit (Zymo Research Corporation, Irvin, CA, USA), following the manufacturer’s instructions. First strand cDNAs for 3′ and 5′ rapid amplification of cDNA end (RACE) were synthesized by SuperScript II reverse transcriptase (Thermo Fisher SCIENTIFIC, Waltham, MA, USA) attached to the 3′ and 5′ RACE system (Thermo Fisher) from total RNA. The first polymerase chain reaction (PCR) was performed using GSPF1 (5′-^260^ATGACTACGTTCTTATCAAA^279^-3′ coding ^38^MTTFLIK^44^), an abridged universal amplification primer (AUAP), and RTase as a template. The nested PCR was performed with GSPF2 (5′-^280^AGTGGAAAGTTTCTACCA^297^-3′ coding ^47^SGKFLHP^53^) as a forward primer and with AUAP as a reverse primer. cDNA was synthesized starting from total RNA and was subjected to 35 cycles of PCR amplification (95 °C for 30s, 42 °C for 30 s and 72 °C for 1 min) in a C1000™ thermal cycler apparatus (Bio-Rad, Hercules, CA, USA). The final elongation step was carried out at 72 °C for 10 min. The 3′ RACE PCR product was subcloned into a pGEM-T easy vector (Promega, Madison, WI, USA) and the sequence was determined using a BigDye Terminator v3.1 cycle sequencing kit (Applied Biosystems, Weiterstadt, DE, USA) and an ABI 3130 genetic analyzer (Applied Biosystems Inc., Foster City, CA, USA). The first strand cDNA for 5′ RACE was synthesized from total RNA with RTase using the sequence-specific primer GSPR1. To create the 5′ RACE abridged anchor prime (Thermo Fisher) binding site, the tailing reaction was performed with terminal deoxynucleotidyl transferase (Thermo Fisher) and dCTP as a substrate. The first PCR to amplify the 5′ RACE PCR product was performed with GSPR1 and AUAP using the dC-tail cDNA as a template. The nested PCR was performed with GSPR2 and AUAP with the same conditions mentioned above. The nucleotide sequence obtained by 3′ and 5′ RACE covering the coding region was identified as the full-length cDNA of MytiLec-1. The result was confirmed by RT-PCR amplification using primers GSPF3 (5′-^139^GCACTATAAATGACTGCTGGT^159^-3′) and AUAP with a PrimeSTAR Max DNA polymerase (TaKaRa Bio Inc., Ohtsu, Japan). The amplified DNA was cloned and sequenced as described above.

### 3.2. Transcriptome Analysis of Full Length of MytiLec-1 mRNA

We used the cDNA sequence of MytiLec-1 obtained as described above to screen the *de novo-*assembled transcriptome data of *M. galloprovincialis* obtained from multiple sources, including: (i) the digestive gland from a mussel population from the Gulf of Trieste [40]; (ii) gills from a mussel population from the Goro lagoon (Northern Adriatic Sea, Italy); (iii) mixed tissues from mussel populations from Sete (Mediterranean Sea, France), Gibraltar (Mediterranean Sea, Spain) and Roscoff (France, English channel) [41]; (iv) gills, posterior adductor muscle, hemocytes, and mantles from a mussel population from Vigo (Atlantic Sea, Spain) [42]; and (v) anterior, mid and posterior mantles from a mussel population from Ria Formosa (Atlantic Sea, Portugal) [43]. These RNA-seq datasets were imported in the CLC Genomics Workbench 8 environment (Qiagen, Hilden, Germany), trimmed based on quality, and *de novo* assembled as described in Gerdol *et al.* 2014 [40].

The assembled transcript corresponding to the putative MytiLec-1 sequence identified by PCR as described in Section 3.1 was detected by BLASTn. The correct assembly of the MytiLec-1 transcript consensus was checked by back-mapping RNA-seq reads to the sequence and by a manual assessment of a uniform and homogenous mapping along the entire coding sequence. The expression level of MytiLec-1 in different tissues was calculated as RPKM (read per kilo-base per million mapped reads) with the CLC Genomics Workbench RNA-seq mapping tool (CLC Bio, Aarhus, Denmark) setting the length and similarity fraction parameters to 0.75 and 0.98, respectively, and match/mismatch/deletion penalties to 3/3/3. The RNA-seq datasets considered for this analysis were digestive gland [40], gills, hemocytes, posterior adductor muscle [41], anterior, mid and posterior mantle [43].

### 3.3. Gene Structure Analysis of MytiLec-1

The raw genomic DNA sequencing reads of *M. galloprovincialis* derived from the bioproject PRJNA262617 were downloaded from the NCBI SRA database. Reads were assembled with the *de novo* assembly tool of CLC Genomics Workbench 8 and the contig comprising the MytiLec-1 gene was identified by BLASTn using the transcript sequence as a query. The correctness of the assembled genomic contig was evaluated by inspecting paired-end reads coverage and the location of the two exons and of the intron was determined by aligning the MytiLec-1 transcript to the genomic contig with MUSCLE [44]. The correct position of donor and acceptor splice sites was refined with by NNSPLICE v. 0.9 [45]. The presence of a putative promoter region was investigated in the region near the putative transcription start site by NNPP v. 2.2 [46].

### 3.4. Purification of MytiLec-1

Mantles and gills of *M. galloprovincialis* were homogenized in 10 volumes (*w*/*v*) of 150 mM NaCl containing 10 mM Tris-HCl, pH 7.5 (TBS). The supernatant (Sup 1) was collected by centrifugation at 27,500× *g* for 1 h at 4 °C. The precipitate was homogenized in 10 volumes (*w*/*v*) of 100 mM d-galactose containing TBS, and the supernatant (Sup 2) was collected as described above. Sup 2 was dialyzed extensively against TBS. Sup 1 and Sup 2 were both applied to a melibiosyl-agarose column (5.0 mL), and the column was washed with TBS until the absorbance of the effluent at 280 nm reached the baseline level. The lectin was eluted with TBS containing 100 mM melibiose [8].

### 3.5. Bacteriostatic Assay of MytiLec-1

The bacteriostatic assay was performed following the protocols by Naganuma *et al.* and Kovalchuk *et al.* [11,47]. Gram-positive bacteria (*Bacillus subtilis* and *Staphylococcus aureus*) and Gram-negative bacteria (*Escherichia coli* and *Vibrio parahaemolyticus*) were grown overnight in LB Broth (Lennox) (Sigma-Aldrich Co. Ltd., St. Louis, MO, USA) under standard conditions. After harvesting, bacteria were washed with PBS and 50 μL of the bacterial suspension (with a turbidity of 0.7 at 600 nm) in PBS (phosphate buffer saline) was used in a quantitative assay by mixing with a serial dilution of MytiLec-1. The bacterial agglutination was monitored by a light microscope. The washed bacterial suspension was adjusted to an absorbance of about 1.0 at 600 nm. MytiLec-1 was mixed with the bacteria at final concentrations of 10, 100, and 200 μg/mL in a flat-type 96-well microtiter plate and grown at 37 °C. The absorbance at 600 nm was monitored every 4 h. The value of the growth suppressive activity was calculated with the following equation:

Growth suppressive activity (%) = (1 − OD_600experiment_/OD_600control_) × 100%

OD_600experiment_ and OD_600control_ are the density of a bacterial suspension grown in the presence of MytiLec-1 measured at OD_600_ and density of a control bacteria suspension grown in the absence of MytiLec-1 determined at OD_600_, respectively.

## 4. Conclusions

We defined, for the first time, the presence of a novel structural family termed “mytilectin” in the mussel *M. galloprovincialis*. The members of this lectin family (MytiLec-1, -2, and -3) have quite specific primary structures. They display two types of protein conformations: MytiLec-1 only consists of a glycan-binding domain, whereas MytiLec-2 and -3 possess both a glycan-binding domain and a pore-forming aerolysin-like domain. We characterized the MytiLec-1 sequence both at the cDNA and at the genomic DNA level, evidencing that the mRNA is produced by a gene composed by two exons and one intron and that this gene is mainly expressed in the mantle and gill tissues. Although full length cDNA of MytiLec-1 does not encode a classical signal sequence, the virtually-translated ORF indicates the possible presence of an alternative N-terminal extension sequence of 37 amino acids which could provide an alternative non-classical secretion signal. However, in absence of experimental data concerning the use of the first ATG codon as a translation start site, the production of this longer variant of the polypeptide remains hypothetical. The functional overlap and specificity of action of the three mussel mytilectins remain issues to be investigated in the near future.

Lastly, we demonstrated the bacteriostatic activity of MytiLec-1. Altogether these results suggest that MytiLec-1 can function as a pattern recognition receptor molecule in the mussel’s innate immunity.

## Figures and Tables

**Table 1:** Bacteriostatic activity of MytiLec-1 ^a^.

Bacteria	Growth Suppressive Activity (%)	Agglutination ^b^
Gram-positive		
*Bacillus subtilis*	74 ± 8	++
*Staphylococcus aureus*	61 ± 3	+
Gram-negative		
*Escherichia coli*	58 ± 5	++
*Vibrio parahaemolyticus*	67 ± 1	++

^a^ Concentration of MytiLec-1 is 20 μg/mL; ^b^ ++ and + mean strong and good, respectively.
